# Supplementary material for: Diversity, spatial distribution and activity of fungi in freshwater ecosystems
Source: PeerJ. 2019 Feb 21;7:e6247. doi: 10.7717/peerj.6247 (PMC6387782; doi:10.7717/peerj.6247)
Supplement: Figure S1 [file peerj-07-6247-s001.pdf]

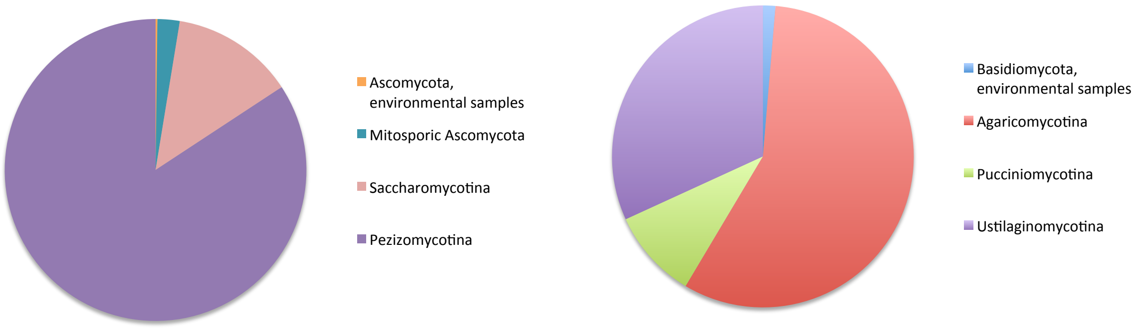

Supplementary figure 1: Taxonomic identities of Ascomycota (a) and Basidiomycota (b) OTUs in freshwater
