# Supplementary figures and images for: Diversity, spatial distribution and activity of fungi in freshwater ecosystems
Source: PeerJ. 2019 Feb 21;7:e6247. doi: 10.7717/peerj.6247 (PMC6387782; doi:10.7717/peerj.6247)

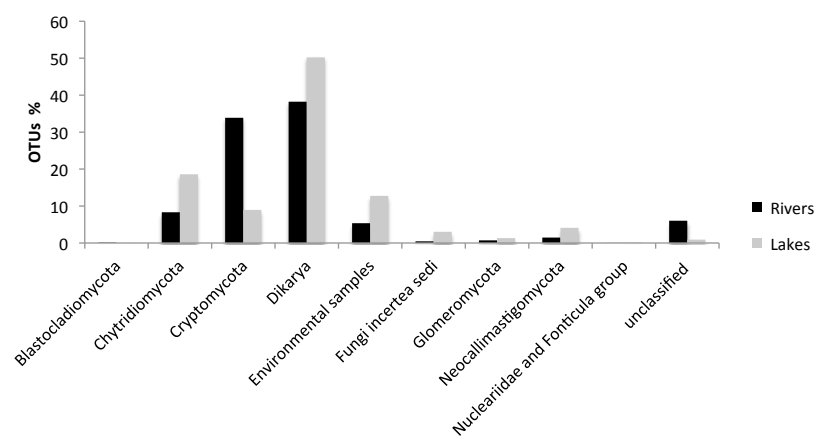

Sup Figure 2: OTUs repartition within the major taxonomic groups in lakes and rivers

Supplement: Figure S2 [file peerj-07-6247-s002.pdf]

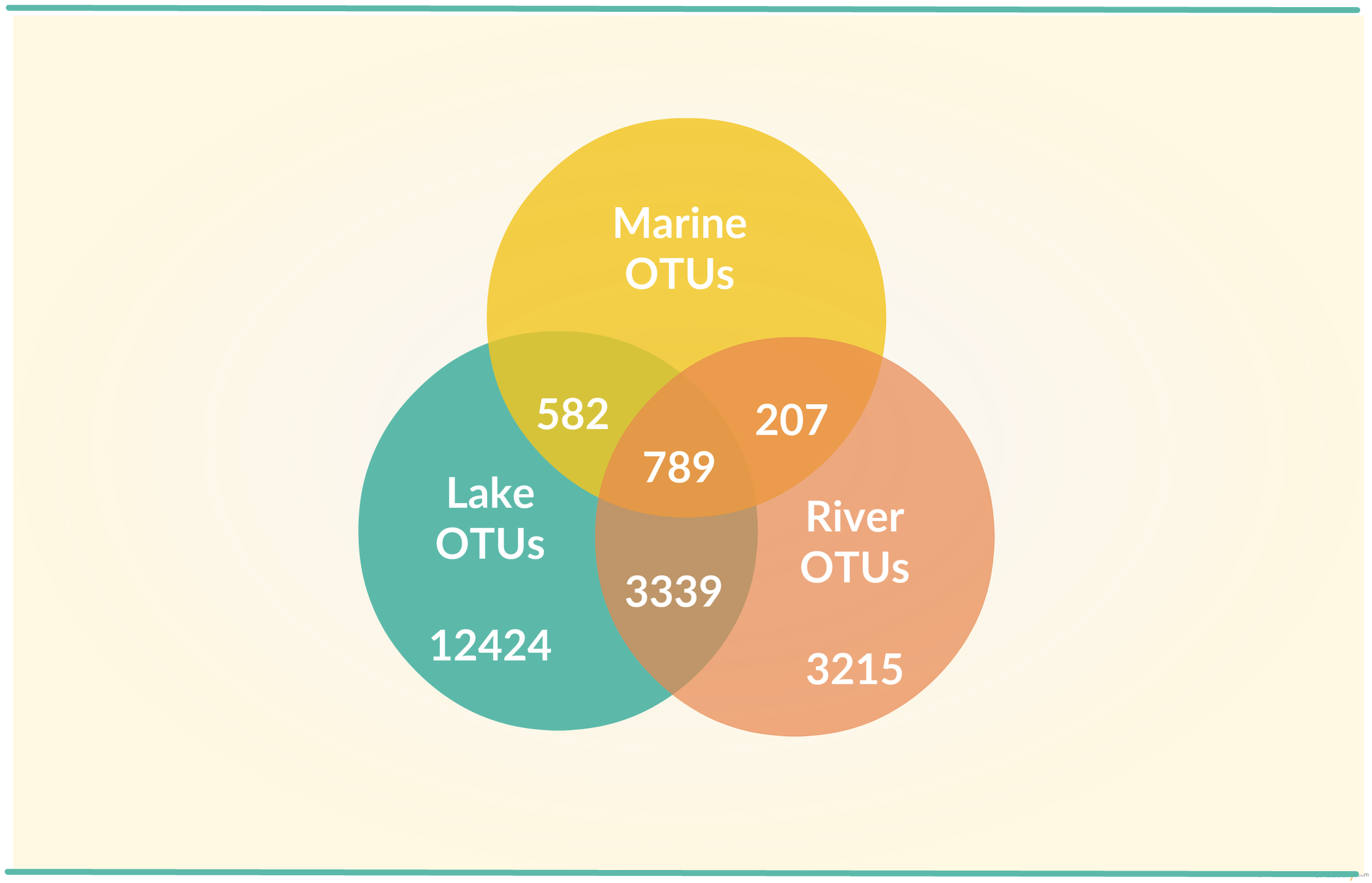

Supplement: Figure S3 [file peerj-07-6247-s003.png]
